# Sexuality and Religious Ethics: Analysis in a Multicultural University Context

**DOI:** 10.3390/healthcare11020250

**Published:** 2023-01-13

**Authors:** Silvia Navarro-Prado, María Angustias Sánchez-Ojeda, Ángel Fernández-Aparicio, María Ángeles Vázquez-Sánchez, Fernando Jesús Plaza del Pino, Inmaculada Alemany-Arrebola

**Affiliations:** 1 Department of Nursing, Faculty of Health Sciences, Melilla Campus, University of Granada, 52005 Melilla, Spain; 2 Instituto de Investigación Biosanitaria (ibs.GRANADA), 18014 Granada, Spain; 3 Department of Nursing, Faculty of Health Sciences, University of Malaga, 29071 Malaga, Spain; 4 Department of Nursing, Faculty of Health Sciences, University of Almería, 04120 Almería, Spain; 5 Department of Developmental and Educational Psychology, Faculty of Education and Sport Sciences, Melilla Campus, University of Granada, 52005 Melilla, Spain

**Keywords:** sexual abstinence, sexuality, young adult, culture, religious ethics

## Abstract

Sexuality is still perceived by some cultures as a taboo subject. Although there is now a more open attitude towards sexuality, the maintenance of virginity is one of the most concerning issues in some religions. The aim of this research is to investigate the sexual behavior of university students and analyze how culture and religion influence the beliefs and maintenance of virginity in women and men. A mixed methodology was used, involving 355 students in the quantitative design and 18 informants for the qualitative study who took part in two focus groups. The results indicate that religion and the degree of religious practice are predictor variables for the decision to have penetrative sex, with Muslim women and men giving more importance to the maintenance of a woman’s virginity. However, this hymen-centric view does not prevent other sexual practices, such as oral and/or anal sex, among young people who wish to maintain their virginity until marriage. Religious ethics continue to influence the sexual behavior of young people today. Therefore, nursing education must address these issues to improve the affective sexual health of the population.

## 1. Introduction

The sexuality of young people has been, and still remains, a subject that has been widely studied across numerous disciplines such as educational, social, and, of course, the health sciences. Sexuality is lived and experienced individually. It is conditioned by numerous aspects such as biological, economic, ethical, political, cultural, and religious factors. Together, these aspects guide people’s sexual behavior [1,2,3], and they must be known to nurses in order to provide quality nursing education [4]. This sexual education should be provided by school nurses [5].

Sexuality is perceived as a taboo subject in many cultural groups, which can lead to a lack of transmission of information about sexuality that results in negative repercussions for the sexual health of young people. Thus, sex education is not accepted by all population groups in the same way. In this sense, there are economic, cultural, political, and religious factors that, in numerous ways, limit, access to information for at-risk populations such as adolescents and young adults [6,7].

Among the religious factors, the existence of a traditionalist view on sexuality in the major religions has been identified, acting as a preventive factor against the early onset of sexual relations, thereby aiding in the preservation of virginity until marriage [8]. Virginity, understood as the absence of sexual relations until marriage, has been considered a fundamental pillar within the most influential religions for centuries [9]. Although the current attitude is more open towards sexuality, the change in values and the modifications to beliefs about virginity have led to changes in the perception of the importance of virginity worldwide [6].

Although sexual relations without marriage or partnership are widely accepted worldwide, there are still societies in which virginity continues to be considered another value for women and a sign of respect for their families and the institution of marriage [10]. Many countries that hold Islamic ideologies continue to be very strict with rules prior to marriage and prioritize the virginity of women. Although these rules extend to both men and women, stigma is greater among the latter [11,12].

This traditional concept of virginity maintenance can cause social and health problems among adolescents. While this stage of development is characterized by increased sexual desire and relationship seeking, the confrontation of tradition, lack of knowledge, and active sexuality can lead to unwanted pregnancies, sexually transmitted infections, hymenoplasty, family conflicts, depression, anxiety, and exclusion from the social environment [13].

Sanabria Mazo et al. [8] found that young Christians tend to be unaware of the norms that Catholicism imposes upon sexuality before marriage. They also tend to separate religious morality and sexual practices more easily than members of other religions. Thus, young believers tend to maintain a more conservative sexual behavior than their less-practicing or non-believing peers, so that religion seems to act as a factor that delays the initiation of sexual relations. 

In contrast, a higher degree of religiosity may negatively influence the use of condoms or other contraceptive methods because they are considered unacceptable from a religious point of view, as they undermine opportunities to increase childbearing [8,14]. 

Apart from the degree of religiosity and in relation to the use of contraceptive methods, there are other factors that influence the use of these methods during first sexual relations in adolescents. These include: the negative view adolescents have of contraceptive methods, considering them unnecessary; a lack of knowledge about contraceptive methods; a lack of access to contraception; the spontaneity of relations; and a lack of trust in sexual partners that hinders the use of contraceptive methods during the first sexual relations [15].

Therefore, the analyzed research indicates that religion and the degree of religiosity are variables that influence the maintenance of virginity. The aim of this research is to learn about the sexual behaviors of young adults regarding the initiation or delay of sexual intercourse, and to analyze the cultural/religious influence on these sexual behaviors.

## 2. Materials and Methods

A mixed methodology was used for this research, since both forms of research provide knowledge that is useful for obtaining more complete information on the research topic under study. A quantitative, ex post facto, exploratory-type study was carried out in combination with a descriptive, qualitative, and inductive research approach to corroborate some ideas relating to the concept of virginity. Intentional, non-probabilistic sampling was used to obtain the sample.

### 2.1. Participants

#### 2.1.1. Quantitative Study

This study was carried out in a frontier city in southern Spain, in which four cultures coexist: Christian Europeans, Jews, Roma, and Muslim, Tamazight-speaking Berbers. The most numerous groups are the Europeans and the Berbers [16], with Muslims representing the 52% of the population (38% natives and 14% Moroccan immigrants) [17]. This research was carried out at an Andalusian university with a total of 355 participants, 81.7% of whom were enrolled in the Degree in Nursing program and 18.3% enrolled in the Degree in Physiotherapy program in the Faculty of Health Sciences. These percentages are representative of the total number of students enrolled in both degrees. The mean age was 21.49 years (standard deviation, SD = 3.24), ranging from 18 to 53 years. Detailing the sample by age range, 275 students were between 18 and 22 years of age (77.5%), 65 students were between 23 and 27 years of age (18.3%), 10 students were between 28 and 33 years of age (2.8%), and only 5 students were over 33 years of age.

In relation to sex, 272 students were women (76.7%) and 83 were men (23.4%), percentages that coincide with the proportion of students enrolled in both degrees. As for the religion with which students identified, 222 considered themselves Christians (62.5%), 79 were Muslims (22.3%), and 54 were agnostics (15.2%). In addition, among the university students who professed some religion, when asked about their degree of religious practice, 129 considered themselves not practicing at all (42.9%), 96 were not very practicing (31.9%), 49 students responded that they were very practicing (16.3%), and 27 were quite practicing (9.0%). The group of students belong to the Islamic religion demonstrated an increase in the percentage of the “very practicing” (76%), compared to 7% of the Christian religion who considered themselves very practicing (17.0%).

In addition, the variable of whether students had a partner at the time of the study was analyzed, with 204 students responding that they did not have a partner (57.5%), compared to 151 (42.5%) who responded that they did. Of this group, 145 considered their relationship to be stable (91.8%). 

#### 2.1.2. Qualitative Study

Regarding the selection of the participants, the key criteria of the focus groups were considered, such as the composition of the group, the characteristics of the participants, and the size of the sample. It was determined that the group was homogeneous in relation to age, sex, religion, and degree of religious practice, thus increasing the opinions, experiences, and perceptions of the subject matter. The sampling carried out was intentional, with the criteria including a capacity for dialogue and listening, and that the informants had an availability of time.

With these considerations met, 18 participants were selected and divided into two focus groups according to sex. Each group was composed of three participants of each religion aged between 19 and 24 years old.

### 2.2. Variables

The study variables were:

- Socio-demographic variables that included age, sex, gender, university degree attended, religion with which participants identified, their degree of religiosity, the presence of a stable partner and the degree of trust held with that partner, and whether participants had penetrative intercourse/coital intercourse;
- Dependent variables that included age at the first instance of penetrative intercourse, use or non-use of a contraceptive method at first intercourse, the reason for not having used a contraceptive method at first intercourse, the type of relationship had with the first person with whom participants had their first instance of penetrative intercourse, and other sexual practices that participants did not consider to have an influence on the maintenance of virginity.
- It should be noted that, when asked about the first instance of penetrative sexual intercourse, participants were asked to reflect on and refer to the intercourse in which they considered that they had lost their virginity: vaginal or anal coitus. Similarly, when asked about other sexual practices, participants were asked to answer about the practices they engaged in that did not interfere with their concept of virginity.

### 2.3. Instrument

#### 2.3.1. Quantitative Study

An adaptation of the Young Adult Sexual Behaviors (YA-SB” questionnaire used and validated by Sanz-Martos et al. [18] to evaluate different aspects of sexuality was used for this study. The questionnaire consists of nine items that include the different sexual practices, with a Likert-type response format including three options: “I do not know what it consists of” (1); “I have not practiced it” (2), and “Yes, I have practiced it” (3). These items were organized into four sections: (i) the level of knowledge about contraceptive methods; (ii) attitudes towards contraceptive use; (iii) sexual activity; and (iv) the level of knowledge about family-planning centers. Therefore, this questionnaire was divided into two main sections: one on sexual behaviors and the other on knowledge, using only the first part for this study. The reliability of the questionnaire on sexual behavior in university students was 0.742.

A section on socio-demographic variables was added to this questionnaire. It included, due to the socio-cultural context of the city in which the research was carried out, subjects’ belonging to different religions/cultures. In this sense, it should be specified that the participants were asked to identify themselves as belonging to one of the religious/cultural groups, so that the researchers did not categorize the participants and they were the ones who identified themselves.

#### 2.3.2. Qualitative Study

The structure of the focus groups was designed to encourage exploratory conversations between the researchers and participants. To avoid being influenced by the opinions of other participants, subjects were asked to write down their concepts of virginity and related aspects. Subsequently, a discussion was opened in each group, delving into the following categories shown in Table 1.

### 2.4. Procedure

#### 2.4.1. Quantitative Study

For the selection of participants, the entire student body of the Faculty of Health Sciences was offered information about the purpose of the study and all those enrolled were offered the opportunity to participate. An informed consent form was signed by all participants. This study belongs to a teaching innovation project awarded by the Quality, Teaching Innovation, and Planning Unit of the University of Granada.

After the students agreed to take part in this research, they were asked to complete a questionnaire with questions related to their sexual behavior. At this point, emphasis was placed on voluntariness and anonymity, as well as on answering with the utmost sincerity. To this end, specific instructions were given on how to complete the questionnaire. Due to the fact that the topics addressed are part of the privacy of individuals, an online modality was chosen for the dissemination and completion of the questionnaire, so that it could be carried out at a place and time where the participants felt the most comfortable.

#### 2.4.2. Qualitative Study

The focus group participants were summoned through institutional mail. Once the day and time were set, a classroom was selected at the Faculty of Health Sciences that met the criteria of having an open space with movable chairs, good acoustics, adequate lighting, and a guarantee of privacy so that the informants felt comfortable and could interact face to face. The duration of the session was approximately two hours. The researchers used a tape recorder, for which prior authorization was requested, to record the sessions. Their subsequent transcription was then carried out.

### 2.5. Data Analysis

#### 2.5.1. Quantitative Study

Statistical analysis was performed using IBM SPSS 26 software (SPSS Inc., Chicago, IL, USA). Descriptive and inferential analyses were performed using parametric contrast analysis, given that the data conformed to normality (Student’s *t*, ANOVA) and correlational analysis. The chi-square test was also used to compare categorical variables. Finally, a regression analysis was performed. A 95% confidence interval was used to detect significance.

#### 2.5.2. Qualitative Study

The qualitative data were obtained through the responses of the focus groups conducted. This methodology allowed us to get closer to the students and an explanatory description of the concept of virginity was obtained. All the opinions of the focus groups were transcribed and reviewed by the three researchers conducting the study. A content analysis of the qualitative data was carried out according to the topic of the research and was divided into the three categories of analysis previously mentioned.

## 3. Results

### 3.1. Quantitative Results

First, we analyzed whether the participants had had penetrative sex; the results indicate that 93 students had not had sex (26.2%), compared to 262 who responded that they had (73.8%). Subsequently, the analyses show that there are no significant differences in having penetrative sex as a function of the variable sex (t = −1.352; *p* > 0.05) but there are significant differences as a function of religion (F = 119.85; *p* < 0.001; η2partial = 0.405) and degree of religiosity (F = 32.375; *p* < 0.001; η2partial = 0.382), with both cases having a large effect size. Multivariate analysis indicates significant differences (F = 5.809; *p* < 0.005; η2partial = 0.048), with agnostic and non-practicing Christian university students reporting that they had had penetrative sex versus very practicing Muslims reporting that they had not, with a large effect size.

Next, focusing on the group of students who responded that they had not had penetrative sex, the results indicate that it was the religious group who defined themselves as practicing Muslims who reported that they did not engage in penetrative sex (69.62%), compared to the Christian, practicing college group (1.35%). Furthermore, the data shows that there were 76 women (27.94% of female respondents) and 17 men (20.48% of male respondents) in the group who had not yet had penetrative sex. In the group of women who had not had penetrative sex, the data indicate that 21 belonged to the Christian religion (12.5% of the Christian women surveyed) and 55 belonged to the Muslim religion (85.93% of women professing the Islamic religion), with no agnostic women found. In the group of men, five identified themselves with the Christian religion (10.6% of the total of Christian men), seven considered themselves Muslims (58.33% of the Muslim university students), and five defined themselves as agnostic (25% of the agnostics). 

In relation to the reasons for not having coital relations, Table 2 analyses the reasons and the sex of these participants.

This line shows the group that responded that they had not had penetrative sexual relations. The sexual practices they had carried out are shown in Table 3 according to sex and religion.

Subsequently, an inferential analysis was performed to determine whether there are differences in the sexual practices of the participants according to the sex variable. The data indicate that there are significant differences (t = 2.49; *p* = 0.015; gHedges = 0.665), with women being those who practiced more touching, masturbation, and oral sex (M = 2.89) and men being those who practiced touching and masturbating (M = 2.00), with a large effect size. In this line, there are also significant differences in relation to the religion variable (F = 25.83; *p* = 0.000; η2partial = 0.365): with agnostics practiced kissing and fondling; those of Christian religion practiced kissing, fondling, masturbation, and touching, and those of Muslim religion practiced oral sex and anal sex (M_Agnostic_ = 1.00; M_Christian_ = 1.69; M_Muslim_ = 3.31), with a large effect size.

Results regarding university students who had had penetrative sex are shown in Table 3. The data indicate that the mean age of first penetrative sex *s* 16.44 years (SD = 1.79) with no significant differences according to the sex variable (t = −0.083; *p* > 0.05), religion variable (F = 0.005; *p* > 0.05), or the degree of religious practice (F = 1.235; *p* > 0.05).

As is shown in Table 3, of the contraceptive methods used during the first instance of coital intercourse, the male condom was the most used among the three religious groups (77.87%). This is followed by no method (9.42%) and reverse (5.74%), in which Muslim men always used barrier methods. In the inferential analysis, no significant differences were observed between the variables sex (t = −0.432: *p* > 0.05), religion (F = 0.865: *p* > 0.05) and degree of religiosity (F = 0.249, *p* > 0.05).

Regarding the reasons for not having used any method for the first instance of penetrative intercourse, the results show that it was because the encounter was an improvised relationship (43.59%) and because participants were too embarrassed to buy contraceptives (20.51%), with no significant differences between the reasons according to gender (t = 0.741; *p* > 0.5), religion (F = 0.221; *p* > 0.05), and degree of religiosity (F = 1.524; *p* > 0.05).

Regarding the relationship that existed with the first person with whom they had sexual intercourse, the data indicate that the majority of participants stated that the person was a partner (64.18%) or friend (29.92%). It was observed in the Muslim religious group that neither women nor men had their first relationship with strangers. The inferential analyses show that there are no differences between women and men (t = −1.059; *p* > 0.05), nor according to the religious group (F = 0.238; *p* > 0.05) or the degree of religiosity of the participants (F = 0.318; *p* > 0.05).

In relation to the sex variable, of the 272 women who participated in the study, 196 had penetrative sex, 62.9% were Christian, 24.6% were Muslim and 12.5% considered themselves agnostic. According to the religious group to which they belong, 100% of the agnostic women affirmed that they have had sexual relations with penetration. This percentage dropped to 87.7% in the group of Christian women, and only 17.9% of the Muslim women affirmed that they had had sexual relations with penetration. In the group of men, of the 83 participants, 55.4% claimed to have had penetrative sex. Of this group, analyzing the sample according to religion, 90.2% belonged to the Christian religious group, 75% were students who considered themselves agnostic, and 41.7% were Muslim. Table 4 shows a profile of the participants who had had sexual relations as a function of the variables sex and the religion they professed.

In addition, linear regression analyses were performed to explore the relationship between the willingness to engage in penetrative sex and the religion of university students. After adjusting the model for the variables—religion and degree of religiosity—a direct association was found between the religion professed (β = −0.198; *p* = 0.000) and the degree of religious practice (β = −0.598; *p* = 0.000) with the willingness to engage in coital sex (Table 5).

### 3.2. Qualitative Results

The following are the research results in terms of the categories analyzed.

In the women’s focus group, opinions and beliefs were divided according to the cultural/religious groups. Thus, for Christian and agnostic women, the concept of virginity is an old and sexist concept.

“it seems to me to be a retrograde concept, which only puts pressure on women and is a burden that many carries unnecessarily.”.

(Christian woman, 19 years old, C1)

For the group of Muslim women, the idea prevailed that virginity is a sign of respect towards a future husband, a way of expressing the purity of the woman, and an important part of the culture.

“I consider that being a virgin at marriage is very important for me, my family and a way of reinforcing and respecting my culture, and yes, of course it is the hymen that matters, any other practice can be done.”.

(Muslim woman, 21 years old, C2)

On one hand, the idea that virginity includes all penetrative sex, whether vaginal, oral, or anal, prevails among Christian and agnostic women. On the other hand, the idea of respecting the integrity of the hymen prevails among Muslim women, who did not give so much importance to other types of penetrative intercourse.

“I have not yet practiced penetrative sex, but not because of any religious conviction, but because I have not found anyone suitable, yes, I think it is silly to classify people as virgins and non-virgins, especially when we are only classified as girls …, I consider that being a virgin is not doing anything at all.”.

(Agnostic woman, 24 years old, C3)

As for the focus group of men, the idea of female virginity and, specifically, of the integrity of the hymen, predominated, with the concept of male virginity considered non-existent because there is no medical evidence that determines the previous existence of penetrative sexual intercourse. It was observed that, within the group of Muslim men, the idea of respect for the woman towards the man prevailed, being essential or very important for the woman to be a virgin at marriage.

“so much modernity does not have to reach the Muslim woman. They respect their husbands by preserving their purity …, … the man can experiment before marriage, in fact, I think it is good for the man to know more about sex to guide his future wife.”.

(Muslim man, 22 years old, C1 and C2)

In contrast, among Christian men, female virginity was not so important and was not considered essential for entering into a relationship.

“virginity is a feminine idea but it is not very important anymore. I don’t go around asking who is or isn’t a virgin, I would be considered a weirdo.”.

(Christian man, 19 years old, C1)

In this line are men who define themselves as agnostic, who report that virginity is not a subject that concerns them and is not something important to them.

“men don’t even look at it, it has never been done. To me, the fact that a woman bleeds the first time she does it is not so important, the truth is, besides, many do not bleed and have been swollen from doing other things that for me are stronger.”.

(Agnostic man, 24 years old, C3)

## 4. Discussion

The aim of this study was to determine the sexual behaviors of young adults regarding the initiation or delay of sexual relations, as well as to analyze the cultural/religious influence on these sexual behaviors. In relation to the sample analyzed, 73.8% had had sexual relations compared to 26.2% who had not, with no significant differences between the sexes, a fact that coincides with the study by Penfold et al. [19]. On the contrary, significant differences were found in those who had not had sexual relations and professed to belong to the Muslim religion. According to Sanabria Mazo et al. [8], young people who profess to belong to the Christian religion tend to be more labile in terms of the norms dictated by their religion in relation to sexuality.

Referring to the age of sexual debut, the young people in this study had their first intercourse at 16.44 years, a mean that coincides with numerous international studies [19,20,21,22,23,24,25,26,27]. Early onset of intercourse is associated with a higher prevalence of risky sexual practices and a higher incidence of contracting sexually transmitted infections and unintended pregnancies [19]. Although the average age of sexual debut has been maintained in recent years, the decision to initiate sex seems to currently be influenced by the media, greater social freedoms, and, above all, the influence of social networks [22,28,29].

The decision to have sex early and/or before marriage is therefore influenced by religion as well as by the degree of religious practice. In fact, the women and men in this study who had not yet had penetrative sex were mostly Muslim, followed by the Christian participants. This data coincides with other studies that demonstrate the influence that a religion has on the sexual practices of its believers, being necessary to initiate them once married [6,10]. In this line, Moral de la Rubia [30] reported that practicing Christians, Buddhists, Jews, and Islamics conceived sex as a couple relationship, while non-believers pointed more to the physical nature of sex. 

Likewise, the degree of religiosity also influences the decision to not have sex, with the most practicing young people mostly delaying the initiation of sexual relations. These results were also reported by Morales [31], who described that mostly young people who consider themselves to be very practicing decided to reserve their sexuality until marriage. In this line, Paul et al. [25] added that being permanently involved in religious activities is one of the predictors of sexual abstinence in young men and women. These data coincide with those obtained in this research, where it was observed that religion and the degree of religious practice are predictors of sexual relations in young people.

In this line, the data obtained in the focus group indicate that the religion and the degree of religious practice are related, with the Christians and agnostics of the focus group, regardless of sex, being among those who considered virginity in women to be unimportant. These data coincide with the results obtained by Saeteros Hernández et al. [22], regarding the lack of existing pressure or social and religious values on men [31]. By contrast, the group of Muslims, including both men and women, did consider the value of virginity in women to be fundamental and a significant element of respect for their own culture, in which sexual relations are accepted within the framework of marriage. This aligns with what was indicated by Valcarcel [32], in which virginity acquires a very important role in religions and especially in the Muslim religion.

This data highlights that the practice of a religion is a protective factor against sexual behaviors that carry risks [31,33]. In this sense, there are currently still societies in which the maintenance of chastity until marriage is taken as a public health strategy, thereby controlling the prevalence of sexually transmitted diseases [6].

The majority of young people who expressed that they had not had penetrative sex based this decision on the fact that they had not found the ideal person, with the second most prevalent reason being the ideals of their culture/religion. This last reason was more frequent among women than among men, and these findings coincide with the study conducted by Paul et al. [25].

Within the group of young people who had not yet had coital relations, it was observed that they engaged in other sexual practices. Women in the present study engaged in touching and masturbation more than men, data that coincide with the research conducted by Saeteros Hernández et al. [22]. However, these data are not in line with the work conducted by Morales [31], who reported that masturbation is more frequent among men. The percentages of touching and masturbation are similar among Christian and Muslim women. Non-believing women did not engage in any other sexual practices. Paradoxically, oral and anal sex were practiced by people professing the Muslim religion, mostly among women. These results were corroborated by the focus group, since the informants considered the concept of virginity to be associated with vaginal penetration, since it is the hymen that must be preserved. As a participant in the study by Mehrolhassani et al. [6] said, “… Not having vaginal sex”, “… for example, I can have anal sex to maintain my virginity.” and, “it means having hymen …”.

Thus, it can be concluded that Muslim women consider the idea of virginity to be centered around the hymen and to be very important, since there are women who even undergo hymenoplasty in order to be able to reach marriage as a virgin. These data highlight the importance that the Islamic faith places on keeping the integrity of the hymen intact until marriage and not contemplating in other sexual practices that also involve penetration, which leads to the performance of certain sexual behaviors in the lives of adolescents and young people [8]. The results obtained in this research coincide with those obtained by Mehrolhassani et al. [6], in which women possessed a hymen-centric concept, justifying the permissiveness of other sexual practices. The women in this study expressed that “it does not mean not having sex. It means having a hymen, which means you can have other kinds of sex.” The studies conducted by de la Rubia [30] and Morales [31] also reported the preservation of vaginal sex while engaging in other penetrative practices such as oral and anal sex, in addition to other practices such as masturbation, viewing pornography, sexual fantasies, or cybersex, among others.

In terms of the contraceptives used by the young people in this study at the first instance of penetrative intercourse, the male condom was the most used in almost all the religious groups, followed by no method and reversal, with hormonal contraceptives being the least used. Women were the ones who used the male condom the most in their first intercourse. These data coincide with the study conducted by Guleria et al. [29] in Northern Europe, in which Danish, Norwegian, and Swedish women chose condoms to prevent unwanted pregnancies and sexually transmitted infections, with the use of hormonal contraceptives being negligible, data that agrees with the present study. In contrast, the results obtained by Gibbs [21] reported the majority use of condoms among men and found an association between contraceptive use at first intercourse and subsequent use.

When asked why they had not used any contraceptive method in their first relationship, improvised intercourse was the reason most often given, followed by embarrassment about buying contraceptives. In relation to the sex variable, impromptu intercourse was one of the reasons given mainly among Christian women and among both Christian and Muslim men. These data are similar to those reported by Sanz-Martos et al [18].

Regarding the partner-relationship maintained at the first instance of sexual intercourse, participants in the present study reported that they were mostly part of a stable couple, followed by being friends with the partner, with a minority response of engaging with strangers. These data coincide with reports by Offiong et al. [24] and Spinola et al. [2]. Regarding the couple relationship, no differences were observed between sexes or cultural groups. In contrast, in the study by Gibbs [21], the results indicated that the prevalence of having a stable relationship for the first coital relationship was more frequent among women, while among men casual relationships were more common. He also found that, regardless of sex, those who had a stable relationship used more effective contraceptives for their first instance of intercourse.

The role of the school nurse is essential in educating young people and adolescents. The nurse, as a promoter of health—and in this case, sexual health—must be trained in ethical, religious, cultural, and affective aspects in order to offer quality health education [4,5].

This work has some strengths and limitations. The main strength of this research is that it provides data on sexual behaviors in a multicultural university context. Another strength is its mixed design, including quantitative and qualitative analyses. Among its limitations are the small size of the sample and the focus on only health science students. 

## 5. Conclusions

Human sexuality is influenced by numerous aspects, from ethical, economic, and political factors to individual experiences. Cultural and religious influences are factors that continue to modulate the behavior of those who identify with their moral precepts. Although the concept of virginity has lost strength in more liberal societies, it is still present in some cultural/religious groups in which the preservation of female purity until marriage is a priority. Thus, we continue to observe how practicing young people prefer sexual abstinence. Paradoxically, the concept of virginity among young practitioners only contemplates vaginal sex and, with it, the integrity of the hymen. On one hand, this ancient vision of considering only the virginity of women to be important has caused many to raise their voices and confront the concept of virginity as a legacy of patriarchy. Thus, we observe that there are numerous concepts of what constitutes “real sex”, since performing oral or anal sex is not considered by all young people to be their first sexual intercourse.

On the other hand, we continue to observe how young people face their first instance of sexual intercourse without the necessary tools to face it safely. Early intercourse, lack of use of barrier contraceptives, or casual relationships only highlight the shortcomings of sexual education in schools. It is necessary to implement the information offered to schoolchildren in order to empower young people to make the decision to initiate their sexual life. In this way, we will achieve not only the prevention of sexually transmitted infections and unwanted pregnancies, but also the sexual fulfillment of young people and adults.

Sex education has traditionally focused on avoiding two major problems: sexually transmitted infections and unintended pregnancies. However, important factors such as respect, human sexual response, gender identity, and sexual identity have not been considered, all of which are included in the affective dimension. The sexual education offered by school nurses and other healthcare providers during childhood and adolescence must contemplate affective sexual, ethical, and cultural aspects in order to achieve its objective, which is none other than to promote the affective sexual health of the population.

## Figures and Tables

**Table 1:** Coding of the focus group categories.

Topic of Research	Categories of Analysis	Codification
Virginity	Virginity as female repression	C1
	Virginity as respect for the family	C2
	Other sexual practices allowed for the preservation of virginity	C3

**Table 2:** Reasons of participants who had not had penetrative sex as a function of sex and religion variables.

		I Have Not Found the Ideal Person N (%)	Culture/Religion N (%)	Fear N (%)	Total N (%)
Sex	Woman	45 (48.4)	24 (25.8)	7 (7.5)	76 (81.7)
	Man	14 (15.1)	3 (3.2)	0 (0)	17 (18.3)
Total		59 (63.4)	27 (29.0)	7 (7.5)	93 (100)

**Table 3:** Sexual practices not considered to interfere with the participants’ concept of virginity according to sex and religion variables.

		Kisses and Caresses N (%)	Touching and Masturbation N (%)	Oral Sex N (%)	Anal Sex N (%)	Oral and Anal Sex N (%)	Total N (%)
Women	Christian	8 (8.6)	13 (13.9)	–	–	–	21 (22.5)
	Muslim	5 (5.3)	14 (15.05)	2 (2.1)	23 (24.73)	11 (11.8)	55 (59.1)
	Agnostic	–	–	–	–	–	0
Men	Christian	–	5 (5.3)	–	–	–	5 (5.3)
	Muslim	1 (1.07)	3 (3.2)	–	3 (3.2)	–	7 (7.5)
	Agnostic	5 (5.3)	–	–	–	–	5 (5.3)
Total N (%)		19 (20.4)	35 (37.6)	2 (2.1)	26 (27.9)	11 (11.8)	93 (100)

**Table 4:** Participants who had had penetrative sex according to sex variables and their religion.

Religion	Christian		Muslim		Agnostic		Total
	Woman (N/%)	Man (N/%)	Woman (N/%)	Man (N/%)	Woman (N/%)	Man (N/%)	N (%)
	150 (57.3)	46 (17.6)	12 (4.9)	5 (1.9)	34 (13)	15 (5.7)	262 (100)
Age of first coital intercourse							
Mean	16.45	12	16.92	15.40	15.71	17.07	
Standard deviation	1.971	1.611	2.429	1.114	2.323	0.961	
Minimum	12	12	13	14	14	15	
Maximum	22	19	20	17	19	19	
Level of religiosity							
None	90 (75)	26 (21.7)	3 (2.5)	1 (0.8)	-	-	120 (56.33)
A little	49 (65.3)	18 (24)	6 (8)	2 (2.7)	-	-	75 (35.21)
A lot	9 (60)	1 (6.7)	3 (20)	2 (13.3)	-	-	15 (7.06)
Quite a lot	2 (66.7)	1 (33.3)	0 (0)	0 (0)	-	-	3 (1.4)
Contraceptive methods used at first coital intercourse							
Male condom	114 (60)	32 (16.8)	9 (4.7)	4 (2.10)	18 (9.5)	13 (6.8)	190 (77.87)
Reversal	8 (57.1)	2 (14.3)	1 (7.1)	0 (0)	3 (21.4)	0 (0)	14 (5.74)
Hormonal contraceptive	5 (71.4)	2 (28.6)	0 (0)	0 (0)	0 (0)	0 (0)	7 (2.87)
None	10 (43.5)	5 (21.7)	1 (4.3)	0 (0)	5 (21.7)	2 (8.7)	23 (9.42)
Condom and hormonal contraceptive	4 (40)	2 (20)	1 (10)	1 (10)	2 (20)	0 (0)	10 (4.1)
Reason for not using contraception at the first coital intercourse							
Improvised relationship	6 (35.3)	5 (29.4)	2 (11.8)	0 (0)	4 (23.5)	0 (0)	17 (43.59)
Feeling ahsamed to buy contraceptives	5 (62.5)	1 (12.5)	0 (0)	0 (0)	2 (25)	0 (0)	8 (20.51)
To avoid making a bad impression	4 (57.1)	0 (0)	0 (0)	0 (0)	2 (28.6)	1 (14.3)	7 (17.95)
To avoid diminishing pleasure	5 (71.4)	1 (14.3)	1 (14.3)	0 (0)	0 (0)	0 (0)	7 (17.95)
Relationship with the person at the first coital relation							
Couple	100 (61.3)	26 (16)	7 (4.3)	4 (2.5)	18 (11)	8 (4.9)	163 (64.18)
Friend	38 (50)	18 (23.7)	5 (6.6)	1 (1.3)	9 (11.8)	5 (6.6)	76 (29.92)
Unknown	10 (66.7)	2 (13.3)	0 (0)	0 (0)	1 (6.7)	2 (13.3)	15 (5.9)

**Table 5:** Participants who had had penetrative sex according to sex variables and their religion.

	Unstandardized Coefficient	Standardized Coefficient	Confidence Interval 95% (B)		
	B	β	Lower	Upper	P
Religion	−0.087	−0.198	1.693	1.783	0.000
Level of religiosity	−0.627	−0.598	0.936	1.291	0.000

## Data Availability

The data presented in this study are available on request from the corresponding author. The data are not publicly available so as to maintain the privacy of participants.
